# Stimulation of dendritic cell functional maturation by capsid protein from chikungunya virus 

**DOI:** 10.22038/IJBMS.2020.40386.9558

**Published:** 2020-10

**Authors:** Vu Xuan Nghia, Nguyen Van Giang, Nguyen Xuan Canh, Nguyen Hai Ha, Nguyen Thuy Duong, Nguyen Huy Hoang, Nguyen Thi Xuan

**Affiliations:** 1 Department of Pathophysiology, Vietnam Military Medical University, Ha Dong, Hanoi, Vietnam; 2 Faculty of Biotechnology, Vietnam National University of Agriculture, Gia Lam, Hanoi, Vietnam; 3 Graduate University of Science and Technology, Vietnam Academy of Science and Technology, 18 Hoang Quoc Viet, Cau Giay, Ha Noi, Vietnam; 4 Institute of Genome Research, Vietnam Academy of Science and Technology, 18 Hoang Quoc Viet, Cau Giay, Hanoi, Vietnam

**Keywords:** Chikungunya virus, Cytokine, Dendritic cell, MAPK, NF-κB

## Abstract

**Objective(s):**

Chikungunya virus (ChikV) infection is characterized by persistent infection in joints and lymphoid organs. The ChikV Capsid protein plays an important role in regulating virus replication. In this study, we hypothesized that capsid protein may stimulate dendritic cell (DC) activation and maturation and trigger an inflammatory response in mice.

**Materials and Methods:**

Mice were intraperitoneally injected with capsid protein and examined for changes in immunophenotype in lymph nodes (LNs). Next, DCs were treated with capsid protein or LPS and then expression of maturation markers, cytokine production, and ability to stimulate CD4^+^ T cells in allo-MLR were analyzed.

**Results:**

Injection of mice with capsid protein led to recruitment of myeloid cells and increased activation of T lymphocytes in LNs. Importantly, treatment of DCs with capsid protein prolonged the activation of IKB-α and up-regulated the number of CD11c^+^CD86^+^DCs and release of TNF-α and IL-12p70 as well as reducing DC apoptosis, all effects were abolished in the presence of Bay 11-7082. In addition, IL-2 production was higher by CD4^+^ T cells stimulated with capsid-treated as compared with LPS-induced DCs.

**Conclusion:**

The observations revealed that capsid protein participates in the regulation of NF-κB signaling and maturation of DCs.

## Introduction

Chikungunya viruses (ChikV) are small spherical enveloped viruses causing severe arthritic diseases on a global scale, therefore threaten public health with high mortality. The ChikV-induced diseases are characterized by persistent infection in joints, muscles, lymphoid organs, liver, and brain, and the viral replication occurs in host cells including macrophages (1) and neuronal cells (2). ChikV infection causes tissue damage by predominant infiltration of inflammatory cells and their activations in infected tissues, leading to host cell death (3, 4). The persistence of ChikV RNA is for at least 4 weeks post-infection (5, 6), and susceptibility is age-dependent in mice (7). Signaling pathways including MyD88 and NF-κB (8), TRIF (9), interferon (IFN) (10), and MAPK (11) have been shown to involve in regulating the viral replication and inflammatory response. Interestingly, the development of vaccines against ChikV has been initially successful. Accordingly, an attenuated and vectored ChikV vaccine elicits protective immunity against pathogens in nonhuman primates (12) and DNA vaccination with E1, E2, and capsid genes of ChikV induces the release of high-titer antibodies in mice (13). In humans, two vaccine candidates including MV-CHIK and VRC-CHKVLP059-00-VP have completed successfully phase I clinical trials and sre in the ongoing phase II study (14-16). 

The immune system is composed of special cells, in which dendritic cells (DCs) are the most professional antigen-presenting cells involved in innate immune responses. DCs can capture and process antigens from peripheral tissue to become mature and migrate to the secondary lymphoid organs such as lymph nodes (LNs) (17). Mature DCs are characterized by Ca^2+^ influx (18), leading to increased expression of co-stimulatory molecules and release of inflammatory cytokines and chemokines (17), which play an essential role in the control of T-cell activation. Recent studies indicated that ChikV replication is decreased by inhibiting Cl^-^ channels, which are activated by Ca^2+^ influx (19). Activity of DCs to induce CD8^+^T cell immunity results in inhibition of ChikV infection (20, 21). In addition to DCs, T and B cells are also known not to be sensitive to ChikV infection (20, 22).

Among ChikV proteins, a capsid is the structural protein of ChikV and plays a crucial role in the viral replication (23) that is blocked by using a picolinate organic compound to target this protein (24). Several studies on this protein indicated that treatment of peripheral blood mononuclear cells (PBMCs) with capsid results in slightly increased intracellular levels of IFN-γ and IL-2 in T lymphocytes (25). The persistent expression of capsid antigen is detected in ChikV- infected mouse macrophages at day 30 after infection (4). However, investigation on the regulatory role of DC function by this protein and molecular mechanisms underlying activation of DCs remain undefined. 

The present study has been performed to determine whether capsid protein induces an inflammatory response in DCs and mice. To this end, mice were intraperitoneally (IP) injected with capsid protein and infiltration of different immune cells into and their activation in LNs were analyzed. For *in vitro* experiments, bone marrow-derived DCs (BMDCs) were stimulated with capsid protein or LPS (used as a positive control) and regulatory effect of this antigen on the expression of surface markers, cytokine release, cytokine production in allogeneic mixed leukocyte reaction (allo-MLR) and cell apoptosis were determined. The activity of NF-κB was also assessed in capsid and LPS- stimulated DCs.

## Materials and Methods

***Mice***

Wild type pathogen-free BALB/c mice at the age of 6 to 8 weeks were purchased from Sigma-Aldrich (USA) and housed in a specific pathogen-free facility at the Institute of Genome Research. The animals had free access to food and drinking water. 

***In vivo capsid protein treatment***

Six-week-old male BALB/c mice were injected IP with capsid protein (10 mg/kg body weight) or PBS for control for 48 hr. All capsid-treated and control mice survived and did not suffer from major weight loss, indicating that this dosage of the drug was well tolerated.

***Bone marrow-derived DCs***

BALB/c mice were anesthetized with isoflurane gas and bone marrow cells were flushed out of the cavities from the femur and tibia with PBS. BMDCs were obtained and cultivated as described before (26). In brief, bone marrow cells were washed twice with RPMI-1640 and seeded out at a density of 4 x 10^6 ^cells per 60-mm dish. Cells were cultured for 8 days in RPMI-1640 (GIBCO) containing: 10% fetal calf serum (FCS), 1% penicillin/streptomycin, 1% glutamine, 1% non-essential amino acids (NEAA), and 50 µm β-mercaptoethanol. Cultures were supplemented with GM-CSF (35 ng/mL, Sigma Aldrich) and fed fresh medium containing GM-CSF on days 3 and 6. Nonadherent and loosely adherent cells were harvested after 8 days of culture. Most (80% or more) of the cells expressed CD11c, which is a marker for mouse DCs. Experiments were performed on days 8–10. BMDCs were stimulated with *Escherichia coli* lipopolysaccharide (LPS, 100 ng/ml, Sigma-Aldrich) or capsid protein {2 µg/ml, this protein was purified by Dr. Vu Xuan Nghia as described elsewhere (27)} in the presence or absence of NF-κB inhibitor Bay 11-7082 (10 µM, Sigma-Aldrich).

***Immunoblotting***

DCs (2 x 10^6 ^cells) were pulsed with LPS or capsid protein for 30, 60, or 120 min and then snap-frozen in dry-ice ethanol bath. Cell pellets were thawed on ice and washed twice with PBS, then solubilized in RIPA-1 lysis buffer. Samples were stored at -80 ^°^C until use for Western blotting. Cell lysates were separated by 10% SDS-PAGE and blotted on nitrocellulose membranes. The blots were blocked with 5% nonfat-milk in triethanolamine-buffered saline (TBS) and 0.1% Tween-20. Then the blots were probed overnight with anti-phospho (p)-ERK, anti-ERK1/2, anti p-p38, anti-p38, anti p-IkBα, anti-IkBα, anti-Bcl-2, and anti-GAPDH (all from Santa Cruz) antibodies diluted in 5% milk in PBS and 0.1% Tween-20, washed 5 times, probed with secondary antibodies conjugated with horseradish peroxidase for 1 hr at room temperature, and finally washed 5 times. Antibody binding was detected with the enhanced chemiluminescence kit (GE healthcare). 

***Immunostaining and flow cytometry***

Isolated LN leukocytes and BMDCs were analyzed by flow cytometry (FACSAria Fusion, BD Biosciences) as described (26). Cells (4 x 10^5^) were incubated in 100 µl FACS buffer (PBS plus 0.1% FCS) containing fluorochrome-coupled antibodies to CD45, CD3, CD4, CD8α, CD19, CD11c, F4/80, CD68, CD11c, CD86, CD69, CD25 and CD11b (all from eBioscience) at a concentration of 10 µg/ml. After incubating with the antibodies for 60 min at 4 ^°^C, the cells were washed twice and resuspended in FACS buffer for flow cytometry analysis.

***Cytokine quantification in cell culture supernatant***

DCs were stimulated with LPS (100 ng/ml) or capsid protein (2 µg/ml) in the absence or presence of NF-κB inhibitor Bay 11-7082 (10 µM) for 24 hr. Cell culture supernatant was collected and stored at -20 ^°^C until use for ELISA. TNF-α and IL-12p70 concentrations in DC culture supernatants were determined by using ELISA kits (eBioscience) according to the manufacturer’s protocol.

***Allogenic mixed leukocyte reaction***

CD4^+^ T cell isolation: spleens of Swiss mice were minced gently and digested with collagenase IV at 37 ^°^C to separate splenic cells and then filtered by 70-micrometer cell strainers. Red blood cells were next lysed with erythrocyte lysis buffer. The splenic cells were stained with a biotin-antibody cocktail for 5 min and subsequently anti-biotin microbeads for 10 min to attain untouched CD4^+^ T cells from single-cell suspensions by using the mouse naive CD4^+^ T cell isolation kit (Miltenyi Biotech).

Allogenic mixed leukocyte reaction (allo-MLR) was determined as previously described (28) with slight modifications. In brief, allo-MLR was performed using mature BMDCs to activate CD4^+^ T cells. The CD4^+ ^T cells were washed twice with PBS and cultured in 96-well plates alone or with BMDCs of BALB/c mice (1:10 DCs to T cells, 10^5^ cells/100 μl) at 37 ^°^C/5% CO_2_ for 5 days. To evaluate cytokine production (IL-2, IL-12p70, and IFN-γ), the cell culture supernatants were collected at day 5 and subjected to ELISA by using ELISA kits (eBioscience) according to the manufacturer’s protocol. 

***Phosphatidylserine translocation and PI incorporation***

To discriminate necrotic/late apoptotic from early apoptotic cells, the presence of phosphatidylserine (PS) on the outer surface of the apoptotic cells was detected from FITC-conjugated annexin V binding to PS at the cell surface and necrosis/late apoptosis was assessed from the amount of PI-positive cells. In brief, 4x10^5^ cells were harvested and washed twice with annexin washing buffer (AWB, 10 mM Hepes/NaOH, pH 7.4, 140 mM NaCl, 5 mM CaCl2). The cell pellet was resuspended in 100 µl of annexin-V-Fluos/PI labeling solution (eBioscience), incubated for 15 min at room temperature. After washing with AWB, the cells were analyzed by flow cytometry (FACSAria Fusion, BD Biosciences)*.*

***Statistics***

Data are provided as means ± SEM, *n* represents the number of independent experiments. Differences were tested for significance using Student’s unpaired two-tailed *t*-test or ANOVA, as appropriate. *P*<0.05 was considered statistically significant.

## Results

***Changes in immunophenotype in capsid-treated mice***

**Figure 1:**
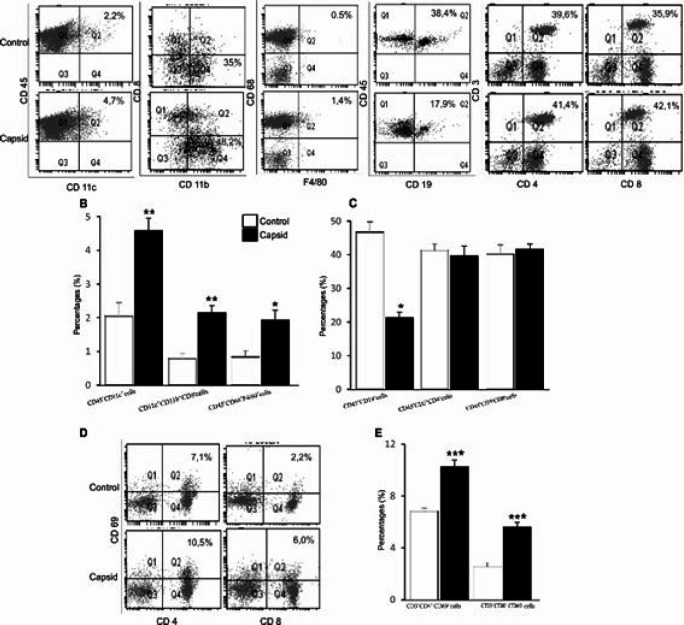
Effect of capsid protein on immune cell infiltration into mouse LNs

Mice were sacrificed 48 hr after IP injection with capsid protein and then LNs were collected for experiments. Infiltration of immune cells into and their activation in LNs were determined by flow cytometry. Upon injection, numbers of myeloid cells including macrophages (CD45^+^CD68^+^F4/80^+^), DCs (CD45^+^CD11c^+^), and myeloid DCs (CD11c^+^CD11b^+^CD8^-^) were expanded, whereas the percentage of B cells (CD45^+^CD19^+^) was reduced in LNs of capsid-treated mice. In addition, numbers of lymphoid DCs (CD11c^+^CD11b^-^CD8^+^), CD4 T (CD45^+^CD3^+^CD4^+^), and CD8 T cells (CD45^+^CD3^+^CD8^+^) in LNs were similar in both groups of mice (Figures 1A-C). Next, enhanced activation of T lymphocytes in LNs of capsid-injected mice was also observed as the expression of CD69 (Figures 1D-E), but not CD25 (data not shown) on CD4, and CD8 T cells was significantly up-regulated.

***Capsid protein prolonged activation of IκB-α in BMDCs***

**Figure 2:**
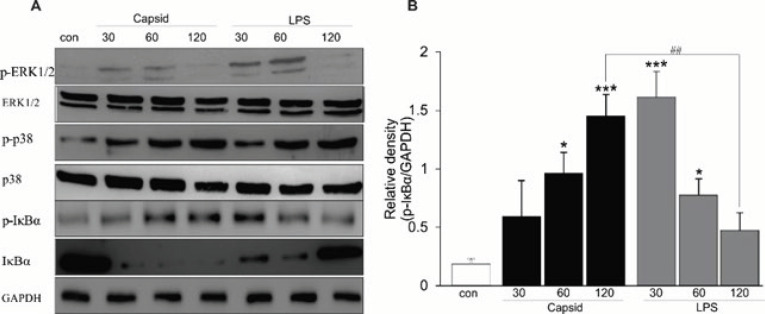
Capsid protein prolonged the phosphorylation of IκB-α in DCs

Recent studies revealed that NF-kB and MAPK signaling pathways participate to modulate the release of inflammatory cytokines and viral replication in ChikV-infected cells (8, 11). In this study, BMDCs were treated with the positive control (LPS) or capsid protein, and total cell protein was extracted by using RIPA-1 lysis buffer. Consistently, stimulation of the cells with LPS or capsid protein resulted in activations of MAPK (p38 and ERK1/2) and IκB-α signalings, however, enhanced phosphorylation of IκB-α in capsid-treated DCs was prolonged for more than 2 hr (Figures 2A-B), whereas that in LPS-stimulated DCs was enhanced and intermediately degraded within 30 min (26). The difference in NF-kB activation between LPS- and capsid-treated DCs would be evidence of persistent ChikV infection.

***Capsid protein up-regulated DC maturation through NF-kB signaling***

**Figure 3:**
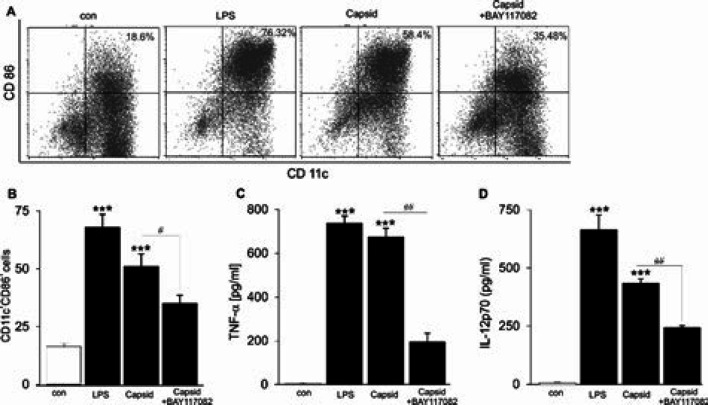
Effect of capsid protein on DC maturation

To ask whether NF-kB activation participates in regulating capsid-induced DC function, immature DCs were stimulated with capsid protein or LPS in the presence or absence of pharmacological inhibition of NF-kB with Bay 11-7082 for 24 hr. Afterward, the cells were harvested and expression of co-stimulatory molecule CD86 (Figures 3A-B) was measured by flow cytometry. Similar to LPS-mature DCs, capsid-treated DCs had significantly higher expression of this maturation marker if compared with untreated DCs, the effect was attenuated by using Bay 11-7082 (Figures 3A-B). We next determined the production of inflammatory cytokine by capsid-treated DCs. As shown in Figures 3C-D, stimulation of DCs with capsid protein resulted in enhanced IL-12p70 and TNF-α cytokines and the effects were inhibited by the presence of Bay 11-7082, suggesting that NF-kB signaling contributed to the stimulating effect of capsid protein on activation of DCs.

***Ability of capsid-stimulated DCs to induce cytokine production in allo-MLR***

**Figure 4:**
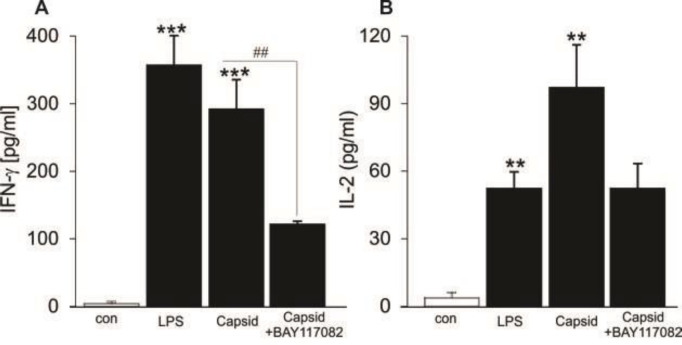
Effect of capsid-stimulated DCs to induce cytokine productions in allo-MLR with CD4^+^ T cells

We next evaluated cytokine production in the culture of allo-MLR. Capsid- or LPS-treated DCs were cocultured with splenic CD4^+^ T cells at a DC: T cell ratio of 1:10 for 5 days and levels of IL-2 and IFN-γ cytokines were analyzed. As illustrated in Figures 4A-B, both LPS- and capsid-stimulated DCs led to cytokine production in allo-MLR, levels of IFN-γ and IL-2 were significantly higher in cell supernatants of T cells stimulated with either LPS- or capsid-treated DCs as compared with control DCs. However, capsid-treated DCs were much more powerful in stimulating IL-2 production than LPS-treated DCs (Figures 4B), indicating that capsid protein may lead to a predominant T cell response.

***Capsid protein inhibited DC apoptosis***

**Figure 5:**
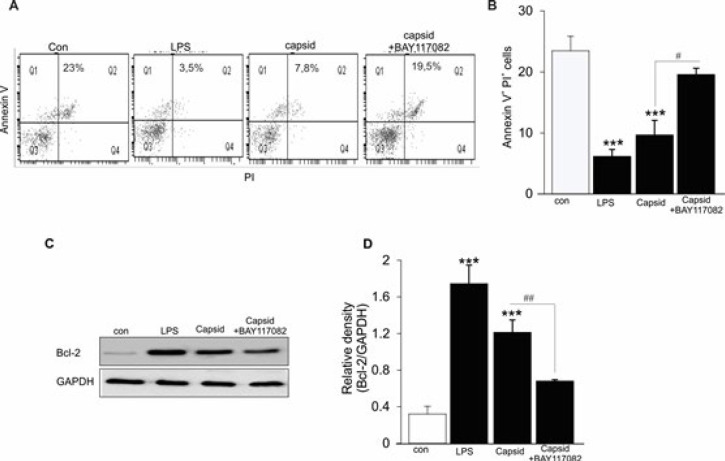
Effect of capsid protein on DC apoptosis

Since mature DCs undergo suicidal cell death, additional experiments were performed to examine the effect of capsid on early apoptotic and necrotic/late apoptotic DC death. Cell membrane scrambling leads to PS exposure at the cell surface whereas PI binding points to late apoptosis/necrosis, thus annexin V^+^/PI^+^ cells are considered as late-stage apoptotic cells. As shown in Figures 5A-B, exposure of mouse DCs to LPS or capsid reduced the number of necrotic cells (annexin V^+^/PI^+^ cells) and unaltered the number of apoptotic cells (annexin V^-^/PI^+^ cells), the effects were abolished in the presence of Bay 11-7082 in the cell culture. In addition, treatment of DCs with capsid resulted in increased abundance of anti-apoptotic Bcl-2, whose expression was inhibited when the cell culture was incubated with Bay 11-7082 (Figures 5C-D). Therefore, the suppressing effect on cell death by capsid was also sensitive to the activation of NF-κB signaling.

A. Original dot plots of CD45^+^CD11c^+^, CD11c^+^CD11b^+^CD8^-^, CD45^+^CD68^+^F4/80^+^, CD45^+^CD19^+^, CD45^+^CD3^+^CD4^+^ and CD45^+^CD3^+^CD8^+^ cells recruited to LNs of mice, which were untreated (control, upper panels) and IP injected with capsid protein (10 mg/kg body weight, lower panels). Numbers depict the percent of cells in the respective quadrants

B-C. Arithmetic means (n=4-6) of percentages of CD45^+^CD11c^+^, CD11c^+^CD11b^+^CD8^-^, CD45^+^CD68^+^F4/80^+^, CD45^+^CD19^+^, CD45^+^CD3^+^CD4^+^ and CD45^+^CD3^+^CD8^+^ cells recruited to LNs of mice that were left untreated (control, white bars) or IP injected with capsid protein (black bars). * (*P*<0.05) and ** (*P*<0.01) represent significant differences from control group, ANOVA

D. Original dot plots of CD3^+^CD4^+^ CD69^+^ and CD3^+^CD8^+^ CD69^+^ cells recruited to LNs of mice, which were untreated (control, upper panels) and IP injected with capsid protein (lower panels). Numbers depict the percent of cells in the respective quadrants

E. Arithmetic means (n=4-6) of percentages of CD3^+^CD4^+^ CD69^+^ and CD3^+^CD8^+^ CD69^+^ cells recruited to LNs of mice, which were left untreated (control, white bars) or IP injected with capsid protein (black bars). *** (*P*<0.001) represents significant differences from the control group, ANOVA

A. DCs were stimulated with LPS (100 ng/ml) or capsid protein (1 µM) for the indicated time: 30, 60, 120 min, or left untreated (control). Protein extracts were analyzed by direct Western blotting using antibodies directed to phosphorylated (p)-ERK1/2, p-p38, p- IκB-α, and IκB-α. Equal protein loading was controlled by total ERK1/2, p38, and GAPDH antibodies. One representative experiment out of four is shown

B. Arithmetic means±SEM (n= 4) of the abundance of phosphorylated IκB-α as the ratio to GAPDH at 0 (control), 30, 60, and 120 min of incubation with capsid protein (black bars) or LPS (grey bars). * (*P*<0.05) and *** (*P*<0.001) indicate significant differences from control, ## (*P*<0.01) shows difference from capsid- or LPS-treated DCs, ANOVA

A. Original dot plots of CD11c^+^CD86^+^ cells, which were untreated (control) or treated with LPS (100 ng/ml) or capsid protein (1 µM) for 24 hr in the presence or absence of NF-κB inhibitor Bay 11-7082 (10 µM)

B. Arithmetic means±SEM (n=5) of percentages of CD11c+CD86+ cells, which were untreated (control) or treated with LPS (100 ng/ml) or capsid protein (1 µM) in the presence or absence of Bay 11-7082 (10 µM) for 24 hr. *** (*P*<0.001) indicates significant difference from control, # (*P*<0.05) denotes difference from capsid-treated DCs, ANOVA

C-D. Arithmetic means±SEM (n=4-6) of TNF-α (C) and IL-12p70 (D) cytokines produced by DCs, which were untreated (control) or treated with LPS (100 ng/ml) or capsid protein (1 µM) for 24 hr in the presence or absence of Bay 11-7082 (10 µM). *** (*P*<0.001) indicates significant difference from control, ## (*P*<0.01) shows difference from capsid-treated DCs, ANOVA

A-B: Arithmetic means±SEM of IFN-γ (A) and IL-2 (B) productions in cell supernatants on day 5 of splenic CD4^+^ T cells co-cultured with DCs untreated (control) or pretreated with LPS (100 ng/ml) or capsid (1 µM) in the presence or absence of Bay 11-7082 (10 µM). ** (*P*<0.01) and *** (*P*<0.001) show differences from control group, ## (*P*<0.01) indicates difference from capsid-treated DCs, ANOVA

A. Original dot plots of annexin V+PI+ DCs, which were untreated (control) or treated with LPS (100 ng/ml) or capsid protein (1 µM) in the presence or absence of Bay 11-7082 (10 µM) for 24 hr. Numbers depict the percent of cells in the respective quadrants.

B. Arithmetic means (n=4) of percentages of annexin V+PI+ DCs, which were untreated (control) or treated with LPS (100 ng/ml) or capsid protein (1 µM) in the presence or absence of Bay 11-7082 (10 µM) for 24 hr. *** (*P*<0.001) represents significant difference from control group, # (*P*<0.05) indicates difference from capsid-treated DCs, ANOVA

C. Protein extracts were analyzed by direct Western blotting using an antibody directed to Bcl-2. Equal protein loading was controlled by the GAPDH antibody. One representative experiment out of four is shown

D. Arithmetic means±SEM (n=4) of the abundance of Bcl-2 as the ratio to GAPDH at 0 (control) and 24 hr of incubation with LPS (100 ng/ml) or capsid protein (1 µM) in the presence or absence of Bay 11-7082 (10 µM). *** (*P*<0.001) indicates significant difference from control, ## (*P*<0.01) shows difference from capsid-treated DCs, ANOVA

## Discussion

In humans, the two vaccine candidates against ChikV are under evaluation in the ongoing phase II study and until now, no FDA-approved vaccines or targeted treatment is available to protect humans from ChikV infection (14-16). In mice, DNA vaccination with envelope E1, E2, and capsid genes of ChikV elicits protective immunity against the pathogen (13, 29), however, the effect of the virus antigens on cellular immune response is little known (23-25). In this study, injection of mice with capsid protein resulted in the infiltration of myeloid cells such as myeloid DCs and macrophages into and activation of T lymphocytes in mouse LNs (Figure 1). In agreement, a recent study indicated that a massive infiltration of myeloid cells and their activation lead to the release of inflammatory cytokines and cytotoxic molecules, causing severe joint inflammation in ChikV-infected mice (9). ChikV replication is cell type dependent as macrophages are sensitive to ChikV infection that is inhibited in the presence of IFN, whereas DCs or T or B lymphocytes prevent ChikV replication (20, 22). Another study indicated that ChikV replication is positively related to virus-infected monocytes (30) and cell activation is induced by viral antigen rather than the toxicity of active viruses (13). In addition, ChikV infection is also limited by activation of Cl^-^ channel (19, 20), which is positively associated with Ca^2+^ influx, a maturation-promoting factor of DCs. Therefore, the stimulating effect of capsid protein on DC biological function in this study is expected to limit ChikV infection and spread. 

Several studies have indicated that molecular mechanisms involved in the regulation of ChikV replication and inflammatory response in different cell types are mediated through several signaling pathways including MyD88 and NF-κB (8), IFN (10) and RIG-I/TBK1/IRF3, but not STAT-1 (31). The phosphorylation of MAPK induced by ChikV infection is linked to functional activation of microglia to produce inflammatory cytokines (11). Consistently, we also showed the role of NF-κB and MAPK signaling pathways in modulating DC function when the cells were exposed to capsid protein. Investigations on the effect of ChikV on functional biology of different subtypes of DCs have been previously reported (21, 29), however, the regulatory roles of DC maturation and differentiation by capsid protein and the signaling molecules involved in the regulation are undefined. In this study, we revealed for the first time that the phosphorylation of IKB-α in capsid-treated DCs was prolonged for more than 2 hr without its degradation (Figure 2), whereas treatment of the cells with LPS leads to phosphorylation and degradation of this protein within 30 min (26). Recent studies reported that the persistent expression of capsid protein in macrophages is observed at 30 days post-infection (4) and chronic ChikV infection in joint-associated tissue in part is due to evasion of antiviral CD8^+ ^T cell immunity (32). The evidence suggested that prolonged activation of NF-κB signaling might influence the T helper (Th)1-Th2 misbalance, resulting in persistence of ChikV infection.

Importantly, the stimulating effect of capsid protein was partially different compared with that of TLR4 ligand (LPS) on DC function. Either LPS or capsid stimulation enhances the expression of co-stimulatory CD86, production of pro-inflammatory cytokines TNF-α and IL-12p70, and induction of CD4^+^ T cell differentiation, as well as reducing cell apoptosis. However, IL-2 release by CD4^+^ T cells stimulated with capsid-treated DCs was detected significantly higher as compared with LPS-treated DCs. Consistently, treatment of ChikV-induced mice with anti-mouse IL-2 antibody reduces joint swelling by inhibiting CD4^+ ^T cell infiltration in draining LNs (33).Contrarily, ChikV-infected macrophages secrete TNF, IL-6, and MCP-1, while the production of IL-10 and IL-12 remain unchanged (3). Although both macrophages and DCs are immune cells responsible for innate responses, they display different phenotypes. Clearly, DCs are CD11c^high^ cells with high antigen-presenting capacity that shape the immune system, whereas macrophages are CD11c^low^ and MHC class II^low ^cells. More importantly, the effects of capsid protein on activation and maturation of DCs were blunted in the presence of pharmacological inhibition of NF-kB with Bay 11-7082 (Figures 2-5), illustrating that the immunomodulatory role of capsid protein was mediated through the activation of NF-κB in DCs. 

## Conclusion

The present study discloses that capsid protein induced inflammatory response in mice and elicited the immunomodulatory effects of DCs mediated through the NF-κB signaling pathway. The events might contribute to efficient prevention or treatment against ChikV infection.

## Ethical Approval

Animal care and experimental procedures were performed according to the Vietnamese law for the welfare of animals and were approved by the ethical committee of the Institute of Genome Research, Vietnam.
